# Vasoactive Intestinal Peptide induces glucose and neutral amino acid uptake through mTOR signalling in human cytotrophoblast cells

**DOI:** 10.1038/s41598-019-53676-3

**Published:** 2019-11-20

**Authors:** Fatima Merech, Elizabeth Soczewski, Vanesa Hauk, Daniel Paparini, Rosanna Ramhorst, Daiana Vota, Claudia Pérez Leirós

**Affiliations:** CONICET - Universidad de Buenos Aires. Instituto de Química Biológica de la Facultad de Ciencias Exactas y Naturales (IQUIBICEN), Buenos Aires, Argentina

**Keywords:** Metabolism, Reproductive biology

## Abstract

The transport of nutrients across the placenta involves trophoblast cell specific transporters modulated through the mammalian target of rapamycin (mTOR). The vasoactive intestinal peptide (VIP) has embryotrophic effects in mice and regulates human cytotrophoblast cell migration and invasion. Here we explored the effect of VIP on glucose and System A amino acid uptake by human trophoblast-derived cells (Swan 71 and BeWo cell lines). VIP activated D-glucose specific uptake in single cytotrophoblast cells in a concentration-dependent manner through PKA, MAPK, PI3K and mTOR signalling pathways. Glucose uptake was reduced in VIP-knocked down cytotrophoblast cells. Also, VIP stimulated System A amino acid uptake and the expression of GLUT1 glucose transporter and SNAT1 neutral amino acid transporter. VIP increased mTOR expression and mTOR/S6 phosphorylation whereas VIP silencing reduced mTOR mRNA and protein expression. Inhibition of mTOR signalling with rapamycin reduced the expression of endogenous VIP and of VIP-induced S6 phosphorylation. Our findings support a role of VIP in the transport of glucose and neutral amino acids in cytotrophoblast cells through mTOR-regulated pathways and they are instrumental for understanding the physiological regulation of nutrient sensing by endogenous VIP at the maternal-foetal interface.

## Introduction

The transport of glucose, amino acids and other nutrients across the placenta involves trophoblast cell specific transporters and its regulation is critical for placental function and foetal growth^1–4^. Deficient or excessive availability of maternal nutrients at the foetal side conditions foetal growth rate and underlie clinical complications like intrauterine growth restriction (IUGR) or large for gestational age newborns. These disorders are associated with perinatal morbidity and with the programming of cardiovascular and metabolic disorders in adult life^5–11^. Likewise, structural and physiological features of the placenta, with altered efficiency at any given stage of pregnancy, predicts a large number of disease

outcomes in adults^9^. Glucose and amino acid uptake capacity of trophoblast cells is highly dependent on the number, density, distribution and activity of transporters^1–3^. GLUT1 and GLUT3 are the main glucose transporters localized on the plasma membrane of the syncytiotrophoblast, a continuous area of nutrient exchange generated by the fusion of the underlying cytotrophoblast cells lining the villi^2^. GLUT1 is considered the primary placental glucose transporter in humans and it is highly expressed in trophoblast cells from the first trimester to term^3^. The uptake of amino acids from maternal circulation is largely mediated by System A and System L transporters expressed by trophoblast cells^1^. System A transports non-essential neutral amino acids against their concentration gradient in a Na^+^-dependent manner (inward Na^+^ gradient) through sodium coupled neutral amino acid transporters SNAT1, SNAT2 and SNAT4, whereas System L mediates essential amino acid uptake by exchanging them with non-essential amino acids driven inside the trophoblast cells by System A^2^.

Accumulating evidence supports a pivotal role for the mammalian target of rapamycin (mTOR) expressed by trophoblast cells in sensing maternal nutrient availability and responding to placental and foetal development requirements^8,12–17^. Trophoblast mTOR activation and signalling is modulated by glucose, amino acid, lipid and folate levels in maternal circulation^8,15,18^, by the supply of oxygen^19^ as well as by hormones and growth factors like insulin, IGF-1 or EGF^8^.

The mechanisms and effectors of glucose and amino acid transport in syncytiotrophoblast cells of term placenta have been extensively reported^2,20–24^. However, little is known about their uptake by cytotrophoblast cells which are particularly relevant in critical processes occurring from the first trimester to term. Indeed, proliferation of cytotrophoblast cells and differentiation to invasive phenotypes, their interaction with vascular smooth muscle or endothelial cells during vessel transformation, and the release of cytokines that condition maternal leukocyte profile to maintain immune homeostasis are all energy requiring processes occurring at placentation^25–27^. Later on, cytotrophoblast cells of human term placenta present greater glycolysis and oxygen consumption than the syncytiotrophoblast *in vitro*^28^, pointing to the central role of cytotrophoblast cells in placental metabolism.

Cytotrophoblast cells synthesize various hormones, growth factors and cytokines that favour foetal growth by targeting nearby syncytiotrophoblast cells, maternal endothelial, stromal vascular and immune cells^25–27^. The vasoactive intestinal peptide (VIP) has been proposed among one of those extracellular cues that regulate placental function and foetal growth based on its effects on immune, vascular and cytotrophoblast cells^29–33^. VIP is synthesized by trophoblast cells and regulates hormone and growth factor release upon binding VPAC1 and VPAC2 receptors^34–37^. VIP has embryotrophic and neurotrophic effects in mouse and rat pregnancy^38–41^. Consistently, VIP deficiency in trophoblast cells but not in maternal tissues adversely affected murine pregnancy outcome with reduced foetal weight, structural placental alterations and immune homeostasis loss^42^. So far, the effect of VIP on nutrient transport in cytotrophoblast cells has not been elucidated.

The main goal of the present study was to explore VIP effect on glucose and System A amino acid uptake by human cytotrophoblast cells. By means of a glucose uptake assay system in single, living cells, we demonstrate that VIP stimulates glucose transport in two human cytotrophoblast cell lines, the first trimester cytotrophoblast-derived Swan 71 cell line and the BeWo cell line. The effect of VIP on glucose uptake was reduced by mTOR inhibition with rapamycin. VIP also stimulated System A amino acid transport and SNAT1 expression. Interplay between VIP and mTOR in nutrient sensing and transport in cytotrophoblast cells is proposed.

## Results

### VIP induces specific glucose uptake in cytotrophoblast cell lines

**Figure 1:**
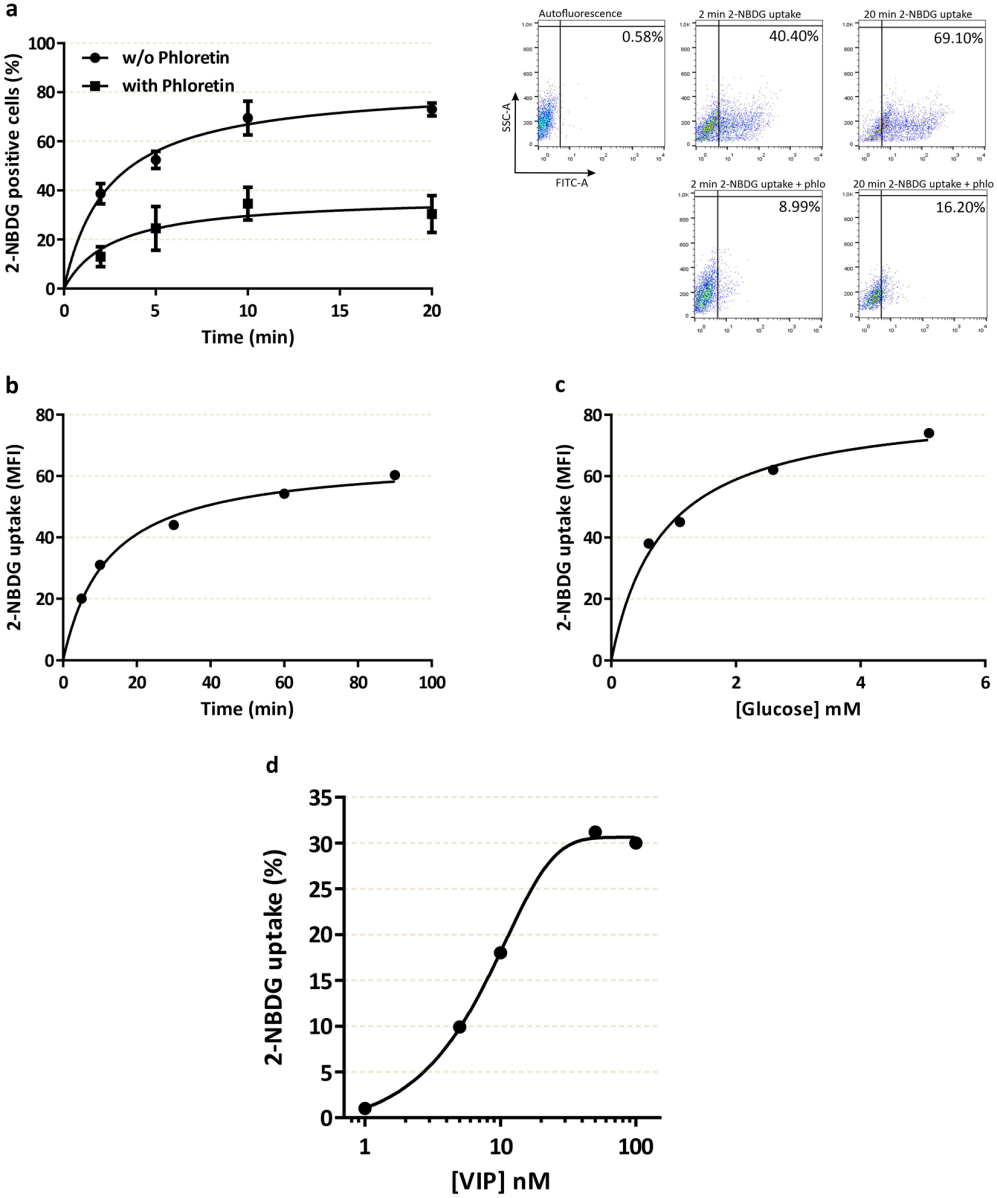
Characterization of 2-NBDG uptake and the effect of VIP in trophoblast cells. Swan 71 cell line was grown until subconfluence, then the cells were washed with cold PBS and medium was replaced by glucose free medium. 2-NBDG was added for different times (**a,b**) or 10 min (**c,d**), cells were washed with cold PBS and flow cytometry was performed. Results in (**b–d**) are express as the difference between without/with 1 mM Phloretin. (**a**) 2-NBDG positive cells at 2, 5, 10 and 20 min in absence/presence of 1 mM Phloretin. Dot plots are representative of autofluorescence control and 2 and 20 min of 2-NBDG incubation in absence/presence of 1 mM Phloretin. Each point represents the Mean ± S.E.M. (**b**) Mean fluorescence intensity (MFI) after incubation with 2-NBDG for 5, 10, 30, 60, and 90 min. The points are representative of 3 different experiments. (**c**) MFI after 10 min incubation with 2-NBDG in glucose free medium supplemented with 0.5, 1, 2.5, 5 mM of glucose. The points are representative of 3 different experiments. (**d**) 2-NBDG uptake by trophoblast cells incubated with 1–100 nM VIP expressed as percent vs. basal value taken as 1. VIP was added 10 min before the addition of 2-NBDG for another 10 min.

**Figure 2:**
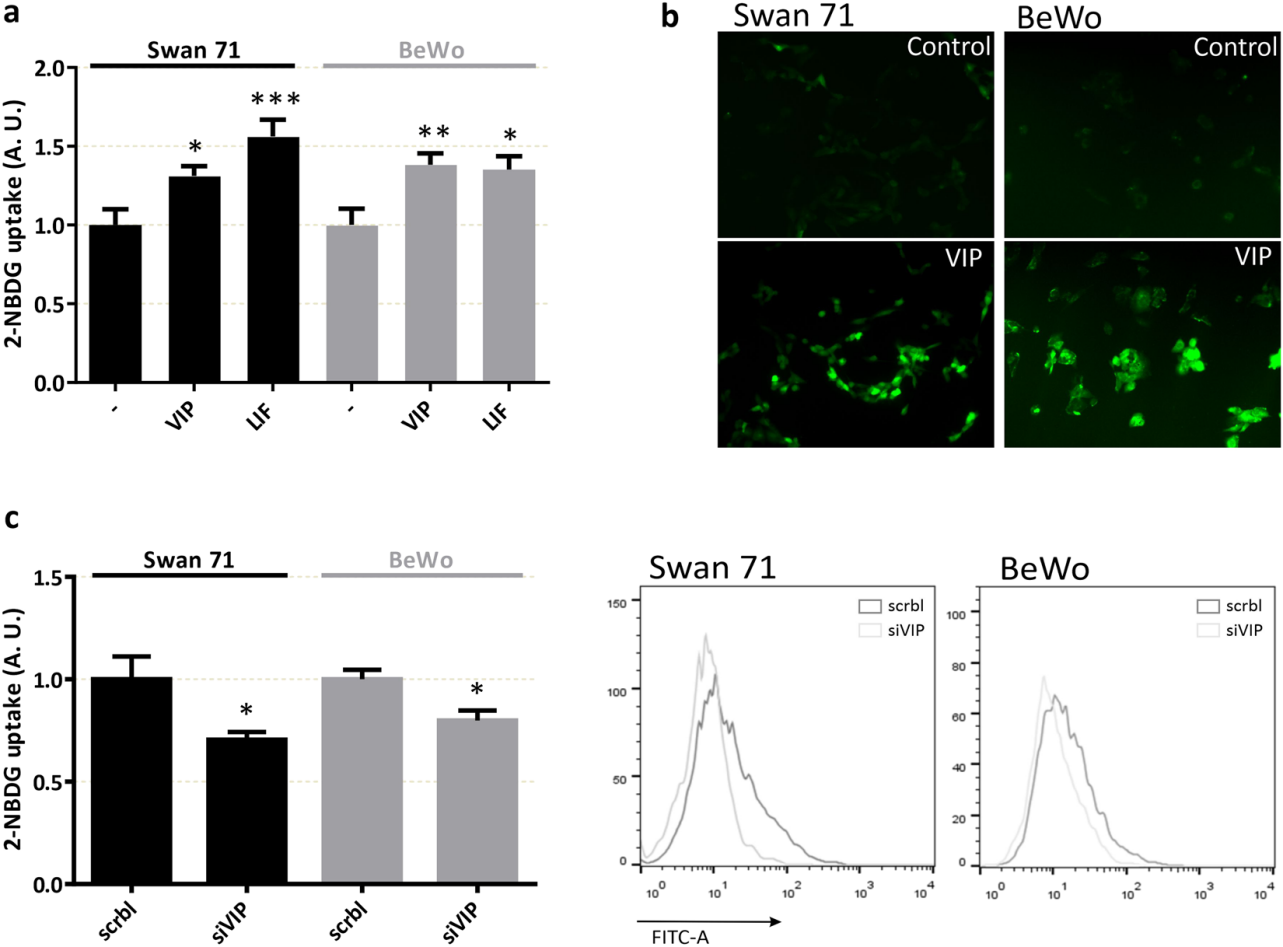
Relevance of trophoblast endogenous VIP for glucose uptake in Swan 71 and BeWo cells. Trophoblast cells (Swan 71 and BeWo cell lines) were grown as in Fig. 1. 2-NBDG was added for 10 min (**a,c**) or 3 min (**b**), cells were washed with cold PBS and flow cytometry was performed. Results are expressed as the difference between without/with 1 mM Phloretin. (**a**) 2-NBDG uptake of cells incubated with 50 nM VIP or 50 ng/ml LIF 10 min before the addition of 2-NBDG (n = 9 for both Swan 71 and BeWo cell lines in VIP treatment and n = 6 in LIF treatment). (**b**) Representative fluorescence microscopy of both cell lines after 3 min incubation with 2-NBDG without/with 50 nM VIP. (**c**) 2-NBDG uptake after VIP was knocked down for 72 h and 2-NBDG were added for 10 min (n = 4). ANOVA with Dunnett’s multiple comparisons against basal condition (**a**) or Student’s t-test was used (**c**). Results are expressed as Mean ± S.E.M. *p < 0.05, **p < 0.01, ***p < 0.001.

We performed a sensitive assay for measuring glucose uptake in single, living cells based on direct incubation of cells with a fluorescent D-glucose analogue, 2-NBDG, followed by flow cytometry detection. The incorporation of the fluorescent probe in the human cytotrophoblast-derived cell line Swan 71 was analysed as the percentage of fluorescent cells (Fig. 1a) or the mean of fluorescence intensity (Fig. 1b,c) in the absence/presence of phloretin, an inhibitor of glucose transport. The assays were carried out at different times or with different concentrations of D-glucose. Figure 1a–c show rapid saturable specific glucose uptake in Swan 71 trophoblast cells. The linearity between 2 and 10 min (Fig. 1b) provided a suitable time of 10 min to evaluate the effect of different stimuli throughout. Next, we investigated the effect of VIP on glucose uptake in human cytotrophoblast cells. As shown in Figure 1d, VIP induced, in a concentration-dependent manner, a significant increase of 2-NBDG uptake in first trimester cytotrophoblast cells. Indeed, the effective concentrations of VIP ranged between 10 and 100 nM so we next assayed 50 nM VIP or 50 ng/ml leukemia inhibitory factor (LIF), a gp130 family cytokine involved in the placentation process^43^ that induces glucose uptake in mouse skeletal muscle^44^. Figure 2a shows that a short incubation with VIP increased 2-NBDG uptake to a similar extent than LIF in both Swan 71 and BeWo cell lines. Consistent with the results of the flow cytometry assays, VIP also increased 2-NBDG incorporation as measured by fluorescence microscopy in both cell lines (Fig. 2b). On the basis that trophoblast cells synthesize VIP^29,35^ and that VIP regulates cytotrophoblast cell function through autocrine loops^29^, loss of function experiments were next carried out to investigate the relevance of endogenous VIP in the regulation of trophoblast glucose uptake. We performed knocking down experiments using a VIP siRNA in Swan 71 and BeWo cells for 72 h, and after confirming the decrease of VIP expression to less than 50% compared to scramble-transfected cells as previously^29,31^, 2-NBDG incorporation was measured by flow cytometry. The results shown in Figure 2c indicate that VIP silencing reduced 2-NBDG uptake in both cell lines suggesting the involvement of endogenous VIP in glucose transport in trophoblast cells.

### VIP increases GLUT1 and GLUT3 glucose transporter expression in cytotrophoblast cell lines

**Figure 3:**
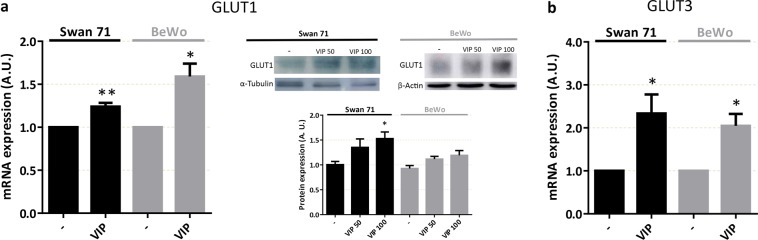
VIP induces the expression of glucose transporters GLUT1 and GLUT3 in human trophoblast-derived cells. (**a,b**) Cells were seeded until subconfluence and 50/100 nM VIP was added for 6 h in DMEM-F12 2% FBS and then cells were harvested. For mRNA analysis, qRT-PCR was performed and results were analysed employing 2^-ΔΔCT^ method normalized to the endogenous GAPDH gene control. For protein analysis, Western Blot was performed and GLUT1 expression was normalized to α-Tubulin or β-Actin. The image shows cropped lines corresponding to α-Tubulin, β-Actin and GLUT1. Blots were run under the same experimental conditions. Full-length gels are presented in Supplementary Fig. S1. Student’s t test or ANOVA with Dunnett’s multiple comparisons against basal condition was used to compare between conditions ((**a**) GLUT1 mRNA: n = 6 for Swan 71 and BeWo; GLUT1 protein: n = 3 for Swan 71 and BeWo; (**b**) GLUT3 mRNA: n = 5 for Swan 71 and n = 4 for BeWo). Results are expressed as Mean ± S.E.M. *p < 0.05, **p < 0.01.

Next, we investigated whether VIP could also regulate the mRNA and protein expression of the main glucose transporters expressed in human placenta GLUT1 (SLC2A1) and GLUT3 (SLC2A3). RT-qPCR, Western blot and flow cytometry assays were carried out in human trophoblast cell lines Swan 71 and BeWo cultured in the absence/presence of 50 or 100 nM VIP for 6 h. As shown in Figure 3a (left and right panel), VIP induced mRNA and protein expression of GLUT1 glucose transporter in both cell lines. GLUT3 mRNA was also induced by VIP, however, no changes were found in GLUT3 expression at the protein level (not shown).

### VIP-induced glucose uptake is mediated through PKA, MAPK, PI3K and mTOR signalling

**Figure 4:**
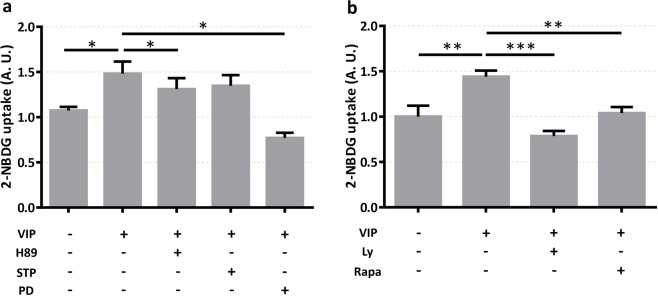
VIP induces glucose uptake through PKA, MAPK, PI3K and mTOR pathways in human trophoblast cells. BeWo cells were cultured as in Fig. 2. Cells were pre-incubated with the corresponding specific kinases inhibitors for 20 min before the addition of 50 nM VIP for 10 min and 2-NBDG for another 10 min. Cells were washed with cold PBS and flow cytometry was performed. (**a**) 2-NBDG uptake in absence/presence of 10 µM H89 (PKA inhibitor), 5 nM STP (PKC inhibitor) or 50 µM PD98059 (MEK inhibitor). (**b**) 2-NBDG uptake in absence/presence of 10 μM Ly294502 (PI3K inhibitor) or 100 nM rapamycin (mTOR inhibitor) (n = 5). RM-one way-ANOVA or ANOVA, with Dunnett’s multiple comparisons test, was used to compare against VIP treatment. Results are expressed as Mean ± S.E.M. *p < 0.05, **p < 0.01, ***p < 0.001.

In order to elucidate the mechanisms involved in the effect of VIP for the regulation of D-glucose transport, we studied the main signalling pathways described downstream VIP activation of its specific receptors VPAC1 and VPAC2: protein kinase A (PKA), protein kinase C (PKC) and MAP kinases (MAPK). 2-NBDG incorporation assays showed that the pre-treatment of trophoblast cells with the PKA inhibitor H89 (10 µM) or the MEK inhibitor PD 98059 (50 µM) prevented the increase of 2-NBDG uptake induced by 50 nM VIP, whereas the PKC inhibitor STP did not prevent VIP effect (Fig. 4a). It has been reported that PI3K induces mTOR complex activation^19,45^ and that mTOR complex is activated by cAMP and would induce the GLUT1 translocation to the plasma membrane^46,47^. Based on this evidence, we analysed the role of PI3K and mTOR activation in the regulation of glucose uptake induced by VIP. As shown in Figure 4b, both the PI3K inhibitor Ly294502 and the mTOR inhibitor rapamycin prevented the increase of 2-NBDG uptake induced by VIP.

### VIP induces amino acid uptake by System A sodium-coupled neutral amino acid transporters (SNAT) in human cytotrophoblast cells

**Figure 5:**
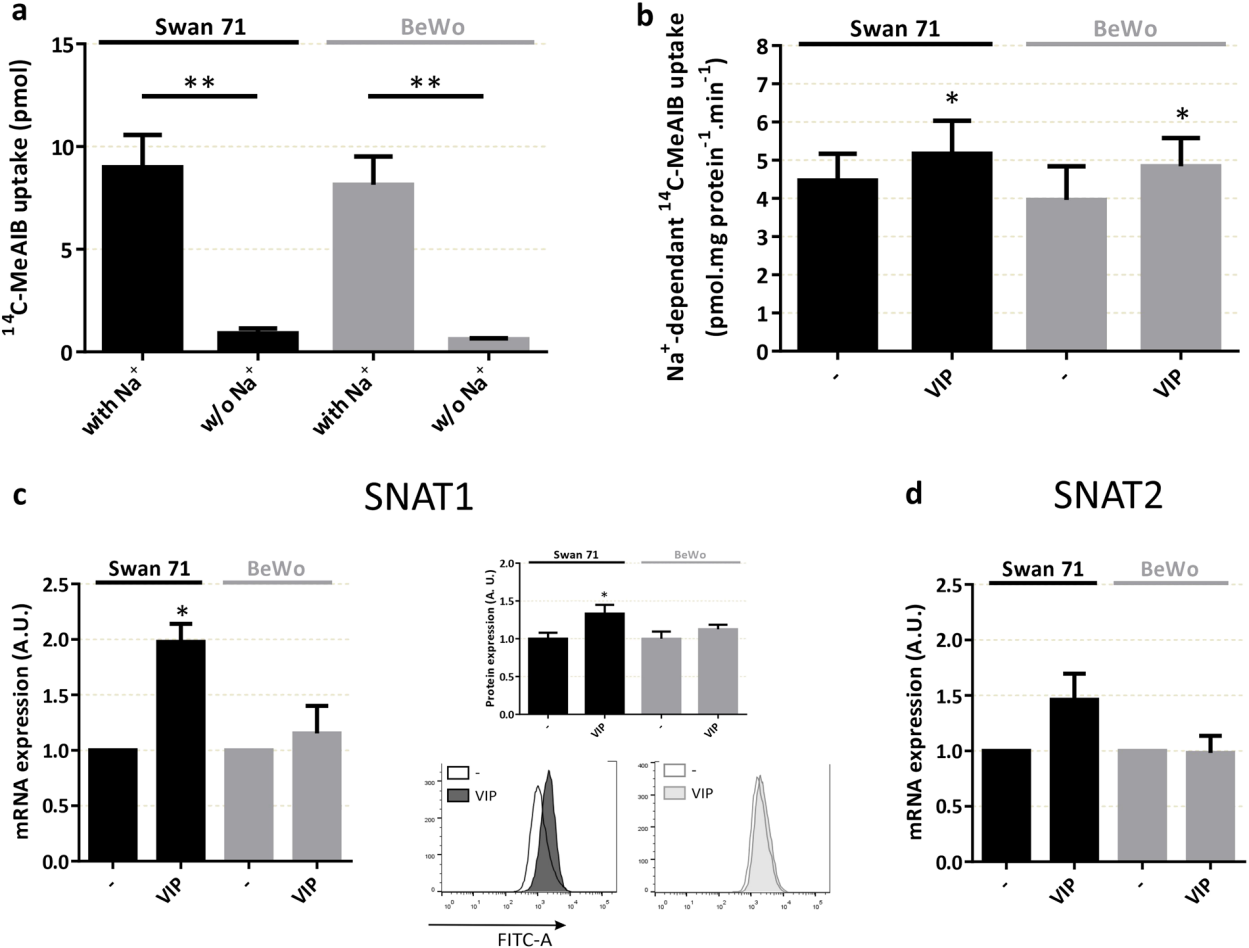
VIP stimulates System A amino acid activity and SNATs expression in human trophoblast cells. (**a,b**) Cells were seeded until subconfluence and then incubated with 50 nM VIP in DMEM-F12 2% FBS for 20 min. Cells were washed with Tyrode’s buffer with/without Na^+^ and ^14^C-MeAIB were added for another 8 min. Cells were washed with cold Tyrode’s buffer without Na^+^ and lysed with distilled H_2_O for 1 h. Liquid scintillation counting was performed. (**a**) Characterization of ^14^C-MeAIB Na^+^-dependant uptake in Swan 71 and BeWo cell lines (n = 4). Results are expressed in pmol ^14^C-MeAIB. **(b**) Na^+^-dependent ^14^C-MeAIB uptake in the absence/presence of 50 nM VIP. Results are expressed as the difference between with/without Na^+^ (n = 4), in pmol ^14^C-MeAIB.mg prot^−1^.min^-1^. (**c**) mRNA and protein expression of SNAT1. Cells were cultured as in Fig. 3 and 50 nM VIP was added for 6 h. Cells were harvested for mRNA analysis by qRT-PCR (n = 4) or protein analysis by flow cytometry (n = 5). (**d**) mRNA expression of SNAT2. Cells were cultured as in (**c**) (n = 7). For mRNA analysis, qRT-PCR was performed and results were analysed employing 2^−ΔΔCT^ method normalized to the endogenous GAPDH gene control. Student’s t test was used to compare between conditions. Results are expressed as Mean ± S.E.M. *p < 0.05, **p < 0.01.

Based on the effect of VIP on glucose uptake and the involvement of mTOR, we next evaluated the effect of VIP on the activity of System A amino acid transporters in Swan 71 and BeWo cell lines. The incorporation of ^14^C-MeAIB, a specific substrate of System A, was measured in sodium-containing and sodium-free (for non-specific transport) Tyrode’s solution (Fig. 5a). System A activity was calculated in all the next experiments by subtracting ^14^C-MeAIB uptake in both conditions. As shown in Figure 5b, VIP induced a significant increase of System A activity in Swan 71 and BeWo cells. Also, VIP increased the expression of SNAT1 but not of SNAT2 mRNA, the two main System A transporters present in human placenta (Fig. 5c,d).

### Cross-regulation between VIP and mTOR in human trophoblast cell lines

**Figure 6:**
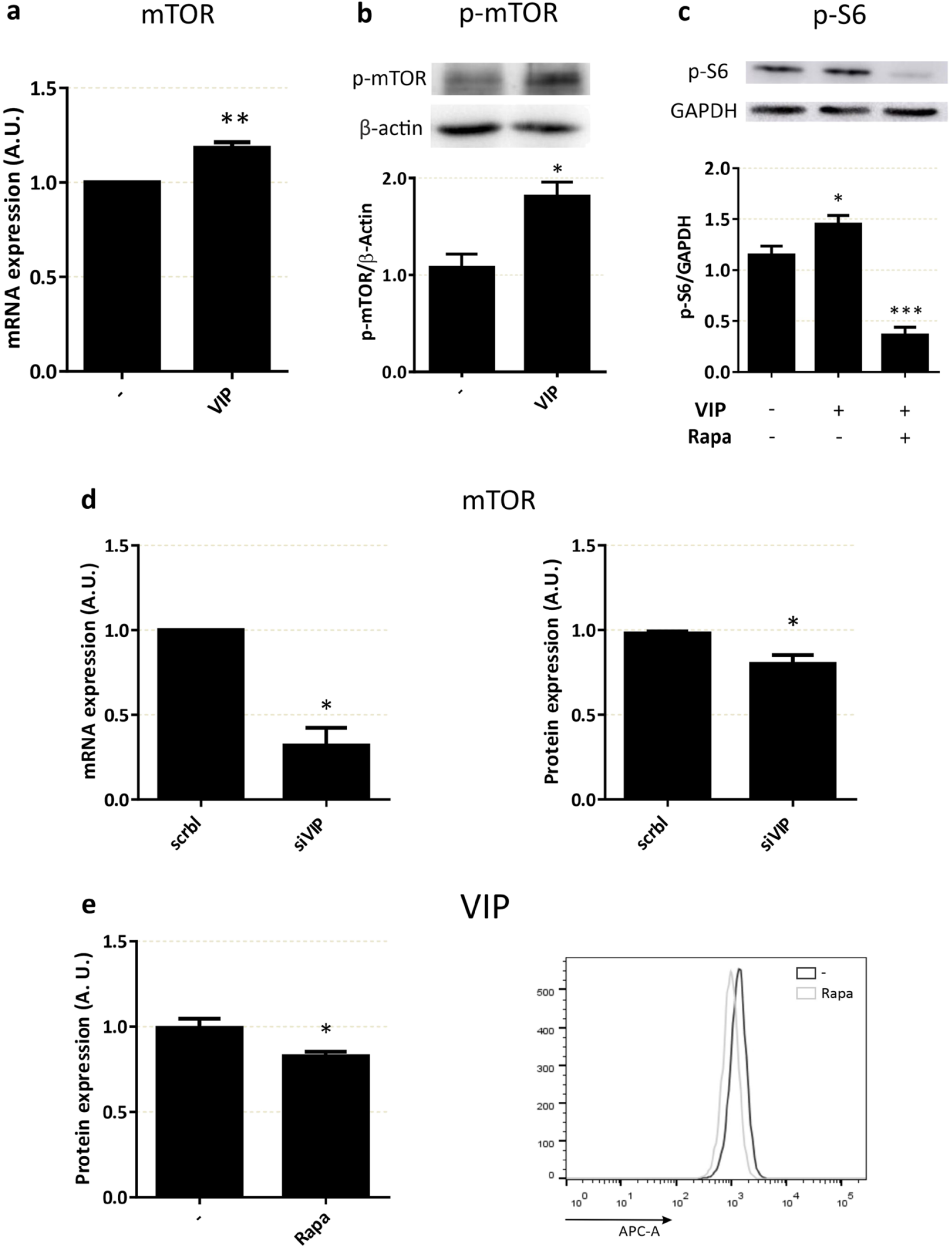
Cross-regulation of mTOR and VIP in trophoblast cells. (**a**) mTOR mRNA expression. Swan 71 cells were seeded and treated as in Fig. 3, in absence/presence of 50 nM VIP (n = 6), qRT-PCR was performed and results were analysed employing 2^−ΔΔCT^ method normalized to the endogenous GAPDH gene control. (**b**) mTOR phosphorylation. Cells were incubated in absence/presence of 50 nM VIP for 20 min, Western Blot was performed and p-mTOR was normalized to β-Actin. The image shows cropped lines corresponding to p-mTOR and β-Actin. Blots were run under the same experimental conditions (n = 4). (**c**) S6 phosphorylation. Cells were incubated in absence/presence of 50 nM VIP for 20 min. 100 µM rapamycin were added 20 min before the stimulus, Western Blot was performed and p-S6 was normalized to GAPDH. The image shows cropped lines corresponding to p-S6 and GAPDH. Blots were run under the same experimental conditions (n = 3). Full-length gels are presented in Supplementary Fig. S1. (**d**) mTOR expression in VIP-silenced cells. Swan 71 cells were seeded, VIP was knocked-down for 72 h and treated as in (**a**) for mRNA expression, or flow cytometry was performed for protein expression (n = 3). (**e**) VIP expression. Cells were cultured as in Fig. 3, then 100 µM of rapamycin was added for 24 h. Protein secretion was inhibited by *Stop Golgi*® incubation for 4 h and cells were subjected to VIP immunostaining and quantification by flow cytometry (n = 3). ANOVA with Dunnett’s multiple comparisons against basal condition or Student’s t test was used to compare between conditions. Results are expressed as Mean ± S.E.M. *p < 0.05, **p < 0.01, *** p < 0.001.

On the basis that the effects of VIP on glucose uptake involved the activity of mTOR and that VIP-knocked down trophoblast cells incorporated less glucose than control scramble-transfected cells, we explored the link between mTOR and VIP in these cells. VIP induced mTOR expression (Fig. 6a) and phosphorylation (Fig. 6b). In line with the involvement of VIP-activated mTOR phosphorylation pathways in cytotrophoblast cells, VIP also induced the phosphorylation of S6, a substrate downstream mTOR activation, and the effect was inhibited by rapamycin (Fig. 6c). A role of endogenous VIP in cross-regulatory loops with mTOR was also tested. VIP-silenced cells presented lower expression levels of mTOR mRNA and protein (Fig. 6d) and conversely, the inhibition of mTOR activity with rapamycin resulted in a reduced expression of VIP (Fig. 6e).

## Discussion

Our results point to a novel role of VIP in the regulation of nutrient transport in cytotrophoblast cells through a mechanism that involves mTOR. The following observations support this proposal: First, VIP increased specific phloretin-sensitive glucose uptake in two human cytotrophoblast cell lines. Deficiency of VIP expression in trophoblast cells resulted in a reduced uptake of glucose whereas VIP induced GLUT1 mRNA and protein expression and GLUT3 mRNA expression in both cells lines. Rapamycin, an mTOR inhibitor, prevented VIP-induced glucose uptake that was also dependent on PKA, MAPK and PI3K but not PKC signalling. Second, VIP increased Na^+^-dependent amino acid transport as well as SNAT1 mRNA and protein expression in cytotrophoblast cells. Third, VIP induced mTOR expression, phosphorylation, and mTOR downstream substrate S6 phosphorylation as well, which was blocked by rapamycin. In parallel, cells silenced in VIP expression presented reduced mRNA and protein expression of mTOR. Moreover, rapamycin reduced VIP expression suggesting that the regulation of VIP levels and VIP-activated signalling is downstream mTOR activation in cytotrophoblast cells.

Cumulative evidence supports the role of VIP as an endogenous regulatory peptide during early pregnancy. In human pregnancy, cytotrophoblast and syncytiotrophoblast cells of first and third trimester placenta express VIP^35^. VIP induces hCG and progesterone release in the human trophoblast JEG-3 cell line and in human trophoblast primary cultures^35^. In the Swan 71 and the HTR8/SVneo human cytotrophoblast cell lines, VIP promotes trophoblast cell migration and invasion through CRE-PKA pathways^29^. In line with an active role of endogenous VIP in cytotrophoblast cell regulation, VIP knocked-down cells presented lower expression of metalloproteases^31^, reduced basal and LIF-mediated migration^29^ as well as they failed to regulate the functional phenotype of maternal leukocytes^32^. Regarding trophic effects of VIP, it displayed an embryotrophic effect on mouse embryos explanted with their yolk sacs at day 9.5^39^ whereas lower birth-weight pups were born from mothers deficient in VIP compared to wild type mice^48^. Also, an association of reduced levels of VIP expressed by trophoblast cells and reduced foetal growth has been recently demonstrated in a VIP deficient murine model^42^. Reduced foetal weight at gestational day 14.5 along with several structural abnormalities of the placenta was reported in pregnancies with VIP-deficient trophoblast cells^42^. Interestingly, the reduced foetal weight observed was not associated with lower placental weight suggesting a metabolic rather than trophic effect of the polypeptide in the placenta^42^.

Based on the multiple energy-requiring functions of the cytotrophoblast cells to support the syncytium, here we tested the hypothesis that VIP contributes to placental metabolism through the modulation of glucose and amino acid uptake by cytotrophoblast cells. Our results clearly support this proposal since VIP increased the transport of glucose and amino acids in two human trophoblast-derived cells lines (Swan 71 and BeWo) that share many characteristics of cytotrophoblast cells and having the BeWo cells the ability to syncytialize. The concentration range of VIP to modulate glucose and amino acid uptake shown here is the same as that recently reported for the migration and invasion of human trophoblast cell lines^29^ as well as for trophoblast migration in primary cultures of human first trimester placental explants^31^. Accordingly, VIP concentrations used here are consistent with VPAC1 and VPAC2 receptor-mediated effects, both subtypes expressed on Swan 71 and BeWo cytotrophoblast cells^31,37^. Moreover, VIP not only induced glucose and amino acid transport but also increased the expression of GLUT1 mRNA and protein and GLUT3 mRNA glucose transporters. Similarly, VIP increased the expression of SNAT1 amino acid transporter isoform. Maternal circulating glucose is the primary energy substrate for foetal and placental growth^3^. Three isoforms of the GLUT family, GLUT1, GLUT3 and GLUT4, are involved in trophoblast glucose uptake, with a higher relevance of GLUT1 and GLUT3 as they are constitutively expressed on trophoblast membranes^24,49^. GLUT1 is the only isoform abundantly expressed in early pregnancy and at term^49^. On the other hand, SNAT1 and SNAT2 activity increases throughout gestation in animal models and in human placenta from first trimester to term^50,51^. Both insulin and Insulin like growth factor 1 (IGF1) have been appointed as the major extracellular signals that promote foetal growth through increasing the transport of glucose and amino acids in trophoblast cells^8,52–54^. The rapid effect of VIP on glucose uptake seems to be direct and not mediated by insulin or IGF1 release since VIP is added only for a 20 minutes time lapse. However, the fact that VIP induced GLUT1 and SNAT1 expression in cytotrophoblast cells as shown here strongly suggests that VIP acting as a growth factor might enable further long lasting adaptive activation of glucose and amino acid transport in trophoblast cells. On the basis that VIP is detected at 2–15 pM concentrations in the serum of pregnant women and in cord blood^55^, it is likely that VIP targets placental cells throughout pregnancy and, despite its short half-life, it might rapidly trigger receptor-mediated signalling that sustain metabolic and trophic functions at the maternal-placental interface.

VIP receptors are coupled primarily to Gs increasing cAMP and activating PKA, with crosstalk signalling in parallel or downstream cAMP that involves NOS^56^, PKC^57^, phosphatidylinositol 3-kinase^58^, MAPK^59–63^, JAK/STAT and NF-kB^64,65^. Our results in cytotrophoblast cells indicate that phosphorylation pathways mediated by PI3K, MAPK, PKA, mTOR but not by PKC activation are involved in VIP-induced glucose uptake. Phosphorylation mediated by PI3K and mTOR is implicated in glucose uptake by brown fat cells and lung adenocarcinoma cells^46,47^. In turn, in BeWo trophoblast cells the transport of glucose induced by resistin involves MAPK-mediated phosphorylation^66^. mTOR integrates multiple hormonal, stress and energy signals involved in foetal and placental growth. Both glucose and amino acid transport are regulated by the mTOR pathway in response to maternal signals like IGF1, insulin and leptin^14,67–69^, all displaying a stimulatory effect, or by inhibiting signals like adiponectin and hypoxia^17,68,70^. In cultured primary human trophoblast cells, the stimulation of System A activity by insulin and IGF1 was shown to depend on mTOR signalling^8^, similar to the stimulatory effect of VIP on glucose uptake shown here for two human trophoblast-derived cell lines.

Finally, a noteworthy observation is that VIP and mTOR signalling appeared mutually regulated in cytotrophoblast cells: VIP induced mTOR expression and mTOR/S6 phosphorylation. Silencing VIP expression down-regulated the levels of mTOR mRNA and protein and conversely, inhibiting mTOR activity with rapamycin reduced VIP protein expression and VIP-induced phosphorylation of S6, a substrate downstream mTOR activation. These observations along with the reduced glucose uptake in VIP silenced cells or upon blocking mTOR and the main VIP signalling cascades strongly support a cross-talk of mTOR and VIP signalling pathways in cytotrophoblast cells. Thus, the levels of VIP expressed by cytotrophoblast cells as well as their responsiveness to VIP stimulation seem to fine-tune the ability of mTOR to integrate multiple signals of the milieu sensed by the cytotrophoblast. In conditions where VIP levels declined or trophoblast cells were less responsive to VIP stimulation, the uptake of glucose and amino acids would be down-regulated whereas mTOR-mediated nutrient uptake would be impaired as well, a scenario observed in several models of IUGR. A link between mTOR and VIP expression has been proposed in suprachiasmatic neurons^71^. Mice deficient in mTOR expression showed a lower expression of VIP and altered susceptibility to constant light stimulation^71^. Likewise, a functional correlation between VIP expression and mTOR signalling was demonstrated in a conditional mTOR knockout mouse model where the mTOR gene was knocked out specifically in VIP-expressing cells^72^. An erratic circadian behaviour, weakened synchronization among cells in the suprachiasmatic nucleus together with reduced olfactory sensitivity was observed in these animals. There are no reports on a relationship between VIP and mTOR in human cytotrophoblast cells, but conclusive evidence indicates that mTOR inhibition impairs trophoblast cell invasion and migration in first trimester Swan 71 trophoblast cells^12^. Decreased migration and invasion is a feature of VIP-knocked down Swan 71 cytotrophoblast cells^29^, the same cells displaying decreased mTOR expression in the present data. Taken together, these observations strongly suggest that VIP regulates cytotrophoblast cell function and nutrient transport through mTOR activation.

To our knowledge, this is the first report on the effect of VIP on nutrient uptake in cytotrophoblast cells. These effects seem to be relevant at all stages of pregnancy considering the various functions of cytotrophoblast cells that require rapid available energy sources early at placentation and beyond as a support for the syncytium. Moreover, the mutual regulation of VIP and mTOR signalling for glucose transport in cytotrophoblast cells shown here supports a central role of the cytotrophoblast to sense maternal nutrient availability and placental demands throughout pregnancy and is in line with recent reported evidence that cytotrophoblast rather than syncytiotrophoblast cells dominate glycolysis and oxidative phosphorylation in human term placenta^28^. Although this is an *in vitro* design and we cannot ascertain that these mechanisms operate *in vivo*, our present observations are instrumental for understanding the role of VIP as one of the molecules implicated in placental metabolism.

## Methods

### Trophoblast-derived cell line cultures

Two human trophoblast-derived cell lines were used. BeWo cell line was derived from human placental choriocarcinoma and Swan 71 cell line obtained by telomerase-mediated transformation of a 7-week cytotrophoblast isolate were kindly given by Prof. Gil Mor (Yale University, New Haven, US). Cells were maintained in culture flasks at 37 °C, 5% CO_2_ in Dulbeco’s modified Eagle’s medium and Nutrient Mixture F12 (DMEM-F12) (Life Technologies, Grand Islands, NY, US) supplemented with 10% heat-inactivated foetal bovine serum (FBS), 2 mM Glutamine (Sigma-Aldrich, Missouri, US) and 100 U.ml^−1^ streptomycin-100 µg.ml^−1^ penicillin solution (Life Technologies, Grand Islands, NY, US)^27,73^.

### VIP silencing

Swan 71 and BeWo cells were transfected with a VIP siRNA (Santa Cruz Biotechnology, Dallas, TX, US) as previously described^29^. Briefly, cells were grown at 60% of confluence and arrested for 3 h in Optimem®. 100 nM VIP siRNA: Lipofectamine RNAimax (Life Technologies, Grand Island, NY, US) complex were made in Optimem and incubated for 15 min prior to addition to the cells in a drop wise manner. siRNA with a scramble sequence was used as a negative control (Scrbl).

### 2-NBDG uptake

2 × 10^4^ Swan 71 or BeWo cells were cultured until subconfluence. Cells were washed with cold PBS and incubated 10 min in glucose free DMEM-F12 medium. For kinetic assays, 100 μM 2-(N-(7-Nitrobenz-2-oxa-1,3-diazol-4-yl)Amino)-2-Deoxyglucose (2-NBDG) (Molecular Probes, Oregon, US) were added for 2–90 min at 37 °C, 5% CO_2_. When VIP was knocked down, 100 μM 2-NBDG was added for 10 min at 37 °C, 5% CO_2_ after the incubation with glucose free DMEM-F12 medium. When stimuli were added, cells were incubated in glucose free DMEM-F12 medium with the stimuli for 10 min prior to the addition of 2-NBDG fluorescent probe. For assays involving signalling pathways inhibitors, 10 µM H89 (PKA inhibitor), 5 nM STP (PKC inhibitor), 50 µM PD98059 (MEK inhibitor), 10 μM Ly294502 (PI3K inhibitor) or 100 nM rapamycin (mTOR inhibitor) were added 20 min before the stimuli. Cells were washed briefly with cold PBS twice, trypsinized, and re-suspended in 2% FBS in PBS to flow cytometry analysis. All assays were carried out in parallel with 1 mM of the glucose transport inhibitor Phloretin (Sigma Aldrich, Missouri, US). Specific glucose uptake was calculated by subtracting the uptake in the presence of Phloretin from the total uptake. The data was acquired in a FACS Aria II cytometer® (Becton Dickinson, San José, CA, US) and was analysed using the FlowJo software (http://www.flowjo.com/). For fluorescence microscopy, cells cultured in the presence/absence of VIP were incubated with 100 μM 2-NBDG for 3 min and photographs were taken immediately.

### Western blotting

Cells were seeded until subconfluence. For protein expression, 50/100 nM VIP were added for 6 h in DMEM-F12 2% FBS and cells were harvested with RIPA buffer supplemented with protease inhibitor cocktail for protein extraction and quantification. For mTOR and S6 phosphorylation assays, 50 nM VIP were added for 20 min in DMEM-F12 2% or 0% FBS. 100 µM rapamycin were added 20 min before the stimuli. Cells were harvested with RIPA buffer supplemented with protease inhibitor cocktail and 1 mM sodium orthovanadate. Western Blotting was performed as previously described^29^ with minor modifications. 30 or 50 µg of protein extract was mixed with Laemmli Sample Buffer 2 × (BioRad, California, US) containing 0.05% β-mercaptoethanol. Samples were subjected to SDS-PAGE electrophoresis (SDS-PAGE Running Buffer: 125 mM Tris, 975 mM glycin, 0,25% SDS, pH 8,3) and electroblotted onto a nitrocellulose membrane (NC) during 1:15 h (transfer buffer: 25 mM Tris, 195 mM glycine, 0.05% SDS, pH 8.3, and 20% (v/v) methanol). NC was blocked by 1 h incubation in Tris buffer saline (25 mM Tris, 137 mM NaCl, 3 mM KCl, pH 7.4) containing 0.1% Tween 20 and 0.5% skim-milk powder. Then, NC was incubated ON at 4 °C with mouse anti p-mTOR (Santa Cruz Biotechnology, Dallas, TX, US), mouse anti GLUT1 (Santa Cruz Biotechnology, Dallas, TX, US), rabbit anti p-S6 (Cell Signaling, Massachusetts, US), mouse anti β-Actin (Santa Cruz Biotechnology, Dallas, TX, US), mouse anti α-Tubulin (Santa Cruz Biotechnology, Dallas, TX, US) or mouse anti GAPDH (Santa Cruz Biotechnology, Dallas, TX, US) monoclonal antibodies. After washing with TBS–0.1% Tween 20, the NC was incubated with anti mouse or anti rabbit horseradish peroxidase-conjugated antibody (ThermoFisher Scientific, Massachusetts, US) for 1 h at room temperature. After washing, specific antibody signals were detected using the enhanced Chemiluminescence system ECL Plus kit and Luminescent Image Analyzer Amersham Imager 600 (GE Healthcare, UK). For stripping, NC was incubated for 15 min at room temperature in Restore Western Blot Stripping Buffer (ThermoFisher Scientific, Massachusetts, US). After washed, NC was blocked again and protocol was carried out as described above. Images were analysed using ImageJ software (NIH, Maryland, US).

### Flow cytometry

4 × 10^4^ Swan 71 or BeWo cells were seeded until subconfluence. 50 nM VIP/100 µM rapamycin were added for 6 h or 24 h respectively in DMEM-F12 2% FBS. For VIP detection, protein secretion was inhibited by Stop Golgi incubation for 4 h. Cells were trypsinized, fixed and permeabilized with the Cytofix/Cytoperm^TM^ plus kit according to manufacturer’s instructions (Becton Dickinson, San José, CA, US). Cells were washed twice and then incubated for 1 h with mouse anti SNAT1 or rabbit anti VIP (Abcam, Cambridge, UK), mouse anti GLUT3 (Santa Cruz Biotechnology, Dallas, TX, US) or rabbit anti mTOR (Cell Signaling, Massachusetts, US) monoclonal antibodies at room temperature. Cells were washed twice and then incubated with secondary antibody anti mouse-Alexa 488/anti rabbit-Alexa 647 (ThermoFisher Scientific, Massachusetts, US) for 40 min at room temperature. After washing twice with permwash buffer and resuspended in FACS solution, ten thousand events were acquired in a FACS Aria II cytometer® (Becton Dickinson, San José, CA, US) and the data was analysed using the FlowJo software (http://www.flowjo.com/).

### qRT-PCR

mRNA expression of nutrient transporters was analysed by quantitative reverse transcription polymerase chain reaction (qRT-PCR) as previously described^29^. Briefly, 4 × 10^4^ Swan 71 or BeWo cells were seeded until sub confluence. Stimuli were added for 6 h in DMEM-F12 2% FBS, then the cells were harvested with TriReagent (Molecular Research Centre, Ohio, US) and total RNA was obtained. 1 µg RNA was treated with DNAasa I following manufacturer’s instructions (Sigma-Aldrich, San Luis, MO, US) to avoid DNA contamination and the samples were reverse transcribed using a MMLV reverse transcriptase, RNAse inhibitor and oligodT kit (Promega Corporation, Madison, WI, US). cDNA was stored at −20 °C for batch analysis. Samples were incubated with SYBR Green PCR Master Mix (Roche, Basilea, Switzerland) and forward and reverse primers (Table 1) in Bio-Rad iQ5 Real-time PCR system. Data was analysed using the threshold cycle (CT) method (2^−ΔΔCT^ method) normalized to the endogenous GAPDH gene control.

**Table 1:** Primers used in qRT-PCR assays.

Gene	Forward (5′ → 3′)	Reverse (5′ → 3′)
hGAPDH	TGATGACATCAAGAAGGTGGTGAAG	TCCTTGGAGGCCATGTAGGCCAT
hGLUT1	ATGGCGGGTTGTGCCATA	ATAGGACATCCAGGGTAGCTGCTCC
hGLUT3	CGAGACCCAGAGATGCTGTAATG	TGGAAAGAGCCGATTGTAGC
hSNAT1	AGCCACCTCTCTACAGAACAC	GTAACTATCACCACCAGAACGC
hSNAT2	CCGCAGCCGTAGAAGAATGA	GCCAGACGGACAATGAGAAGAA
hmTOR	TTTAGCGGTCATGTCAATGG	GTCATAGCAACCTCAAAGCA

### System A amino acid transport activity

^14^C-MeAIB ([14 C]-methyl amino isobutyric acid) uptake assay was carried out to evaluate System A amino acid activity^14,67^. For cell lines assay, 8 × 10^4^ Swan 71 or BeWo cells were seeded until subconfluence. Stimuli were added for 20 min in DMEM-F12 2% FBS, then cells were washed with Tyrode’s buffer with/without Na^+^ (supplemented with choline, for non-specific uptake). ^14^C-MeAIB (PerkinElmer, Massachusetts, US) were added in correspondent Tyrode’s buffer for 8 min in humidified chamber with 5% CO_2_ at 37 °C. Cells were washed with cold Tyrode’s buffer without Na^+^ and 1 mL of distilled water was added for 1 h at room temperature to lyse the cells. Distilled water containing ^14^C-MeAIB was mixed thoroughly with 3 mL of scintillation fluid (optiPhase HiSafe, Perkin Elmer, Massachusetts, US), and counted in scintillation counter (Beckman LS 6500, Fullerton, CA). Using ^14^C-MeAIB standards, uptake was calculated as picomol ^14^C-MeAIB per milligram of protein per minute, relativizing each treatment to protein content measured by Micro BCA Protein Assay Kit (ThermoFisher Scientific, Massachusetts, US). System A activity was determined by subtracting the uptake in Na^+^-free Tyrode’s buffer from the uptake in Na^+^-containing buffer.

### Statistical analysis

Data was analysed using GraphPad Prism 6 software (GraphPad, San Diego, CA, US). Paired or non-paired Student’s t test, one way ANOVA or RM-one way ANOVA with Dunnett’s multiple comparisons were used for parametric analysis, depending on the assay. Results are expressed as Mean ± S.E.M. Differences between treatments were considered significant at p < 0.05.

## Supplementary information

Supplementary Information

## References

[1] Dimasuay KG, Boeuf P, Powell TL, Jansson T (2016). Placental responses to changes in the maternal environment determine fetal growth. Front. Physiol..

[2] Chassen, S. & Jansson, T. Complex, coordinated and highly regulated changes in placental signaling and nutrient transport capacity in IUGR. *Biochim. Biophys. Acta - Mol. Basis Dis*, 10.1016/j.bbadis.2018.12.024 (2019). 10.1016/j.bbadis.2018.12.024 PMC6650384 30684642

[3] Brett KE, Ferraro ZM, Yockell-Lelievre J, Gruslin A, Adamo KB (2014). Maternal–Fetal nutrient transport in pregnancy pathologies: The role of the placenta. Int. J. Mol. Sci..

[4] Gallo LA, Barrett HL, Dekker Nitert M (2017). Review: Placental transport and metabolism of energy substrates in maternal obesity and diabetes. Placenta.

[5] Mahendran D (1994). Na+ transport, H+ concentration gradient dissipation, and system A amino acid transporter activity in purified microvillous plasma membrane isolated from first-trimester human placenta: Comparison with the term microvillous membrane. Am. J. Obstet. Gynecol..

[6] Kuruvilla AG (2008). Altered activity of the system A amino acid transporter in microvillous membrane vesicles from placentas of macrosomic babies born to diabetic women. J. Clin. Invest..

[7] Glazier JD (1997). Association between the activity of the system A amino acid transporter in the microvillous plasma membrane of the human placenta and severity of fetal compromise in intrauterine growth restriction. Pediatr. Res..

[8] Roos S, Lagerlof O, Wennergren M, Powell TL, Jansson T (2009). Regulation of amino acid transporters by glucose and growth factors in cultured primary human trophoblast cells is mediated by mTOR signaling. Am. J. Physiol. Cell Physiol..

[9] Thornburg KL (2016). Biological features of placental programming. Placenta.

[10] Sibley CP, Brownbill P, Dilworth M, Glazier JD (2010). Review: Adaptation in placental nutrient supply to meet fetal growth demand: Implications for programming. Placenta.

[11] Rosario FJ, Powell TL, Jansson T (2016). Mechanistic target of rapamycin (mTOR) regulates trophoblast folate uptake by modulating the cell surface expression of FR-alpha and the RFC. Sci. Rep..

[12] Knuth A (2014). Placenta Growth Factor Induces Invasion and Activates p70 during Rapamycin Treatment in Trophoblast Cells. Am. J. Reprod. Immunol..

[13] Roos S (2007). Mammalian target of rapamycin in the human placenta regulates leucine transport and is down-regulated in restricted fetal growth. J. Physiol..

[14] Roos S, Kanai Y, Prasad PD, Powell TL, Jansson T (2009). Regulation of placental amino acid transporter activity by mammalian target of rapamycin. Am J Physiol Cell Physiol.

[15] Rosario FJ, Kanai Y, Powell TL, Jansson T (2013). Mammalian target of rapamycin signalling modulates amino acid uptake by regulating transporter cell surface abundance in primary human trophoblast cells. J. Physiol..

[16] Mparmpakas D (2010). Expression of mTOR and downstream signalling components in the JEG-3 and BeWo human placental choriocarcinoma cell lines. Int. J. Mol. Med..

[17] Rosario FJ (2012). Chronic maternal infusion of full-length adiponectin in pregnant mice down-regulates placental amino acid transporter activity and expression and decreases fetal growth. J. Physiol..

[18] Rosario FJ, Powell TL, Jansson T (2017). mTOR folate sensing links folate availability to trophoblast cell function. J. Physiol..

[19] Wullschleger S, Loewith R, Hall MN (2006). TOR signaling in growth and metabolism. Cell.

[20] Jansson T (2016). Placenta plays a critical role in maternal–fetal resource allocation. Proc. Natl. Acad. Sci..

[21] Jansson T (2006). Placental Transport and Metabolism in Fetal Overgrowth – A Workshop Report. Placenta.

[22] Jansson T, Powell TL (2013). Role of placental nutrient sensing in developmental programming. Clin. Obstet. Gynecol..

[23] Zhang S (2015). Placental adaptations in growth restriction. Nutrients.

[24] Lager Susanne, Powell Theresa L. (2012). Regulation of Nutrient Transport across the Placenta. Journal of Pregnancy.

[25] Mor G, Aldo P, Alvero AB (2017). The unique immunological and microbial aspects of pregnancy. Nat. Rev. Immunol..

[26] Aplin JD (2010). Developmental cell biology of human villous trophoblast: Current research problems. Int. J. Dev. Biol..

[27] Aplin JD (2006). Trophoblast Differentiation: Progenitor Cells, Fusion and Migration - A Workshop Report. Placenta.

[28] Kolahi KS, Valent AM, Thornburg KL (2017). Cytotrophoblast, Not Syncytiotrophoblast, Dominates Glycolysis and Oxidative Phosphorylation in Human Term Placenta. Sci. Rep..

[29] Vota D (2016). Vasoactive Intestinal Peptide modulates trophoblast-derived cell line function and interaction with phagocytic cells through autocrine pathways. Sci. Rep..

[30] Paparini D (2015). Trophoblast cells primed with vasoactive intestinal peptide enhance monocyte migration and apoptotic cell clearance through αvβ3 integrin portal formation in a model of maternal–placental interaction. Mol. Hum. Reprod..

[31] Paparini Daniel E., Choudhury Ruhul H., Vota Daiana M., Karolczak‐Bayatti Magdalena, Finn‐Sell Sarah, Grasso Esteban N., Hauk Vanesa C., Ramhorst Rosanna, Pérez Leirós Claudia, Aplin John D. (2019). Vasoactive intestinal peptide shapes first‐trimester placenta trophoblast, vascular, and immune cell cooperation. British Journal of Pharmacology.

[32] Calo G (2017). Trophoblast cells inhibit neutrophil extracellular trap formation and enhance apoptosis through vasoactive intestinal peptide-mediated pathways. Hum. Reprod..

[33] Vota D (2017). Progesterone and VIP cross-talk enhances phagocytosis and anti-inflammatory profile in trophoblast-derived cells. Mol. Cell. Endocrinol..

[34] Deutsch PJ, Sun Y, Kroog GS (1990). Vasoactive intestinal peptide increases intracellular cAMP and gonadotropin-a gene activity in JEG-3 syncytial trophoblasts: Constraints posed by desensitization. J. Biol. Chem..

[35] Marzioni D (2005). Placental expression of substance P and vasoactive intestinal peptide: Evidence for a local effect on hormone release. J. Clin. Endocrinol. Metab..

[36] Fraccaroli L (2009). VIP modulates the pro-inflammatory maternal response, inducing tolerance to trophoblast cells. Br. J. Pharmacol..

[37] Fraccaroli L (2015). VIP boosts regulatory T cell induction by trophoblast cells in an *in vitro* model of trophoblast-maternal leukocyte interaction. J. Leukoc. Biol..

[38] Gressens P, Hill JM, Gozes I, Fridkin M, Brenneman D (1993). Growth factor function of vasoactive intestinal peptide in whole cultured mouse embryos. Nature.

[39] Passemard S (2011). VIP blockade leads to microcephaly in mice via disruption of Mcph1-Chk1 signaling. J. Clin. Invest..

[40] Hill JM (1996). Maternal vasoactive intestinal peptide and the regulation of embryonic growth in the rodent. J. Clin. Invest..

[41] Servoss SJ (2001). IGF-I as a mediator of VIP/activity-dependent neurotrophic factor-stimulated embryonic growth. Endocrinology.

[42] Hauk, V. *et al*. Trophoblast VIP-deficiency entails immune homeostasis loss and adverse pregnancy outcome in mice. *FASEB J*. 302 (2019). 10.1096/fj.201800592RR 30204500

[43] Poehlmann TG (2005). Trophoblast invasion: Tuning through LIF, signalling via Stat3. Placenta.

[44] Brandt N (2015). Leukemia inhibitory factor increases glucose uptake in mouse skeletal muscle. Am. J. Physiol. Endocrinol. Metab..

[45] Wieman HL, Wofford JA, Rathmell JC (2006). Cytokine stimulation promotes glucose uptake via Phosphatidylinositol-3 Kinase/Akt regulation of Glut1 activity and trafficking. Mol. Biol. Cell.

[46] Olsen JM (2014). Glucose uptake in brown fat cells is dependent on mTOR complex 2-promoted GLUT1 translocation. J. Cell Biol..

[47] Makinoshima H (2015). Signaling through the phosphatidylinositol 3-kinase (PI3K)/mammalian target of rapamycin (mTOR) axis is responsible for aerobic glycolysis mediated by glucose transporter in epidermal growth factor receptor (EGFR)-mutated lung adenocarcinoma. J. Biol. Chem..

[48] Lim MA (2008). Regardless of genotype, offspring of VIP-deficient female mice exhibit developmental delays and deficits in social behavior. Int. J. Dev. Neurosci..

[49] Baumann MU, Deborde S, Illsley NP (2002). Placental glucose transfer and fetal growth. Endocrine.

[50] Illsley NP (2000). Glucose transporters in the human placenta. Placenta.

[51] Desforges M, Greenwood SL, Glazier JD, Westwood M, Sibley CP (2010). The contribution of SNAT1 to system A amino acid transporter activity in human placental trophoblast. Biochem. Biophys. Res. Commun..

[52] Baumann MU (2014). Regulation of human trophoblast GLUT1 glucose transporter by Insulin-Like Growth Factor I (IGF-I). PLoS One.

[53] Forbes K, Westwood M (2008). The IGF axis and placental function. Horm. Res..

[54] Sferruzzi-Perri AN, Sandovici I, Constancia M, Fowden AL (2017). Placental phenotype and the insulin-like growth factors: resource allocation to fetal growth. J. Physiol..

[55] Ottesen B (1982). Vasoactive intestinal polypeptide and the female genital tract: Relationship to reproductive phase and delivery. Am. J. Obstet. Gynecol..

[56] Murthy KS, Zhang KM, Jin JG, Grider JR, Makhlouf GM (1993). VIP-mediated G protein-coupled Ca2+ influx activates a constitutive NOS in dispersed gastric muscle cells. Am. J. Physiol. Liver Physiol..

[57] Spengler D (1993). Differencial signal transduction by five splice variants of the PACAP receptor. Nature.

[58] Straub SG, Sharp GWG (1996). A wortmannin-sensitive signal transduction pathway is involved in the stimulation of insulin release by vasoactive intestinal polypeptide and pituitary adenylate cyclase-activating polypeptide. J. Biol. Chem..

[59] Toumi F (2004). Vasoactive intestinal peptide induces IL-8 production in human colonic epithelial cells via MAP kinase-dependent and PKA-independent pathways. Biochem. Biophys. Res. Commun..

[60] El Zein N, Badran B, Sariban E (2008). VIP differentially activates 2 integrins, CR1, and matrix metalloproteinase-9 in human monocytes through cAMP/PKA, EPAC, and PI-3K signaling pathways via VIP receptor type 1 and FPRL1. J. Leukoc. Biol..

[61] Barrie AP, Clohessy AM, Buensuceso CS, Rogers MV, Allen JM (1997). Pituitary Adenylyl Cyclase-activating Peptide Stimulates Extracellular Signal-regulated Kinase 1 or 2 (ERK1/2) Activity in a Ras-independent, Mitogen-activated Protein Kinase/ERK Kinase 1 or 2-dependent Manner in PC12 Cells. J. Biol. Chem..

[62] Villalba M, Bockaert J, Journot L (2018). Pituitary Adenylate Cyclase-Activating Polypeptide (PACAP-38) Protects Cerebellar Granule Neurons from Apoptosis by Activating the Mitogen-Activated Protein Kinase (MAP Kinase) Pathway. J. Neurosci..

[63] Lelièvre V (1998). Differential Effects of Peptide Histidine Isoleucine (PHI) and Related Peptides on Stimulation and Suppression of Neuroblastoma Cell Proliferation. J. Biol. Chem..

[64] Delgado M, Ganea D (1999). Vasoactive intestinal peptide and pituitary adenylate cyclase-activating polypeptide inhibit interleukin-12 transcription by regulating nuclear factor κB and Ets activation. J. Biol. Chem..

[65] Delgado M, Ganea D (2000). Inhibition of IFN–Induced Janus Kinase-1-STAT1 Activation in Macrophages by Vasoactive Intestinal Peptide and Pituitary Adenylate Cyclase-Activating Polypeptide. J. Immunol..

[66] Di Simone N (2009). Resistin modulates glucose uptake and glucose transporter-1 (GLUT-1) expression in trophoblast cells. J. Cell. Mol. Med..

[67] Jansson N, Greenwood SL, Johansson BR, Powell TL, Jansson T (2013). Leptin stimulates the activity of the system A amino acid transporter in human placental villous fragments. J. Clin. Endocrinol. Metab..

[68] Jones HN, Jansson T, Powell TL (2010). Full-length adiponectin attenuates insulin signaling and inhibits insulin-stimulated amino acid transport in human primary trophoblast cells. Diabetes.

[69] von Versen-Höynck F, Rajakumar A, Parrott MS, Powers RW (2009). Leptin Affects System A Amino Acid Transport Activity in the Human Placenta: Evidence for STAT3 Dependent Mechanisms. Placenta.

[70] Nelson DM (2003). Hypoxia reduces expression and function of system A amino acid transporters in cultured term human trophoblasts. Am. J. Physiol. Physiol..

[71] Cao R (2013). Translational control of entrainment and synchrony of the suprachiasmatic circadian clock by mTOR/4E-BP1 signaling. Neuron.

[72] Liu D (2018). mTOR signaling in VIP neurons regulates circadian clock synchrony and olfaction. Proc. Natl. Acad. Sci..

[73] Straszewski-Chavez SL (2009). The Isolation and Characterization of a Novel Telomerase Immortalized First Trimester Trophoblast Cell Line, Swan 71. Placenta.

