# Supplementary material for: Vasoactive Intestinal Peptide induces glucose and neutral amino acid uptake through mTOR signalling in human cytotrophoblast cells
Source: Sci Rep. 2019 Nov 20;9:17152. doi: 10.1038/s41598-019-53676-3 (PMC6868285; doi:10.1038/s41598-019-53676-3)
Supplement: Supplementary file 1 — Supplementary Information [file 41598_2019_53676_MOESM1_ESM.docx]

Vasoactive Intestinal Peptide induces glucose and neutral amino acid uptake

through mTOR signalling in human cytotrophoblast cells

Fatima Merech, Elizabeth Soczewski, Vanesa Hauk, Daniel Paparini, Rosanna Ramhorst, Daiana Vota^1^, Claudia Pérez Leirós^1^

^1^ Equal contribution

CONICET - Universidad de Buenos Aires. Instituto de Química Biológica de la Facultad de Ciencias Exactas y Naturales (IQUIBICEN). Buenos Aires, Argentina.


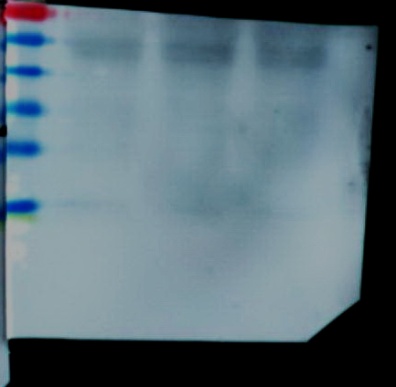

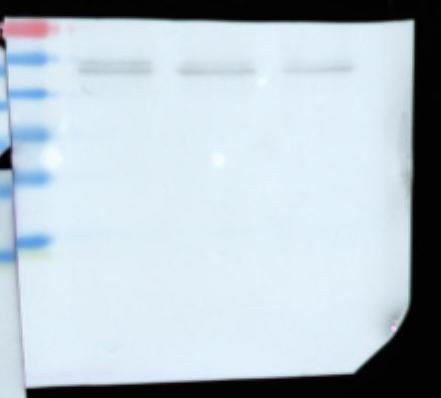


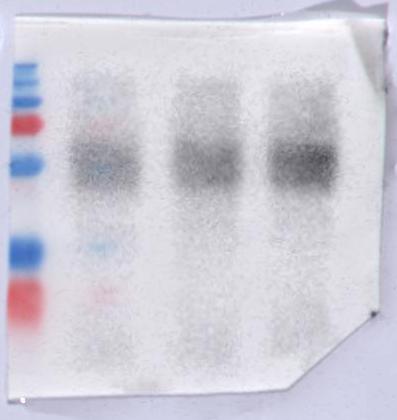

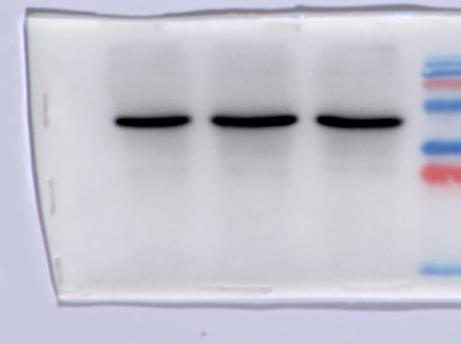


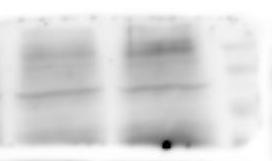

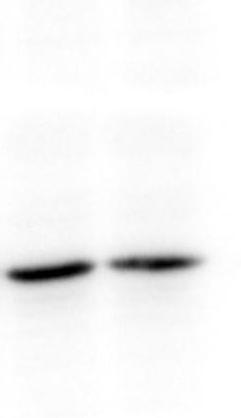


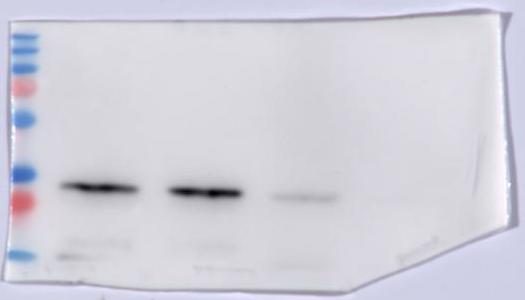

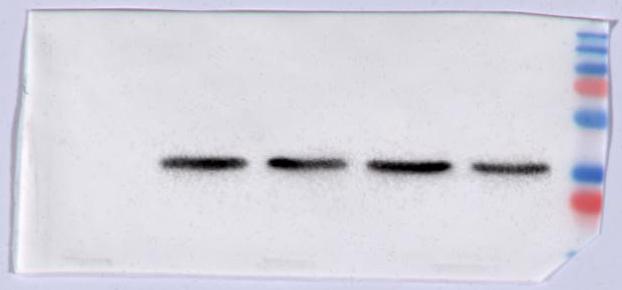


**Supplementary Figure S1**

Full-length gels of cropped gels from Fig. 3 and Fig 6: GLUT1/α-Tubulin from Swan 71, GLUT1/ β-Actin for BeWo, p-mTOR/β-Actin, and p-S6/GAPDH from Swan 71.
